# Tophaceous Gout of the Extensor Mechanism of the Knee

**DOI:** 10.5334/jbr-btr.861

**Published:** 2015-09-15

**Authors:** M. Vansevenant, F. M. Vanhoenacker, F. Catry

**Affiliations:** 1 Department of Radiology, AZ Sint Maarten, Mechelen-Duffel, Belgium; 2 Department of Radiology, Ghent University Hospital (UZ Gent), Belgium; 3 Department of Radiology, Antwerp University Hospital (UZA), Belgium

A 51-year-old male presented at our department with a slightly painful soft tissue lump at the superior pole of the patella and limited range of joint motion. MRI of the left knee confirmed a soft tissue mass within the distal quadriceps extending into the superior aspect of the patella. The mass was hypointense on T1-weighted images (WI) (Fig. A, arrows) and slightly heterogeneous hyperintense on fat saturated intermediate-WI (Fig. B, arrows). A bone infarct was seen in the distal femur (Fig. A and B, arrowhead). After administration of gadolinium contrast heterogeneous enhancement was seen. Gradient-echo sequences did not reveal blooming artifact. Additionally Cone Beam CT showed the soft tissue mass as being slightly hyperdense to the surrounding soft tissue (Fig. C, arrow). Partially sclerotic delineated osteolysis was seen at the patella (Fig. C, arrowhead).

**Figures A–C:**
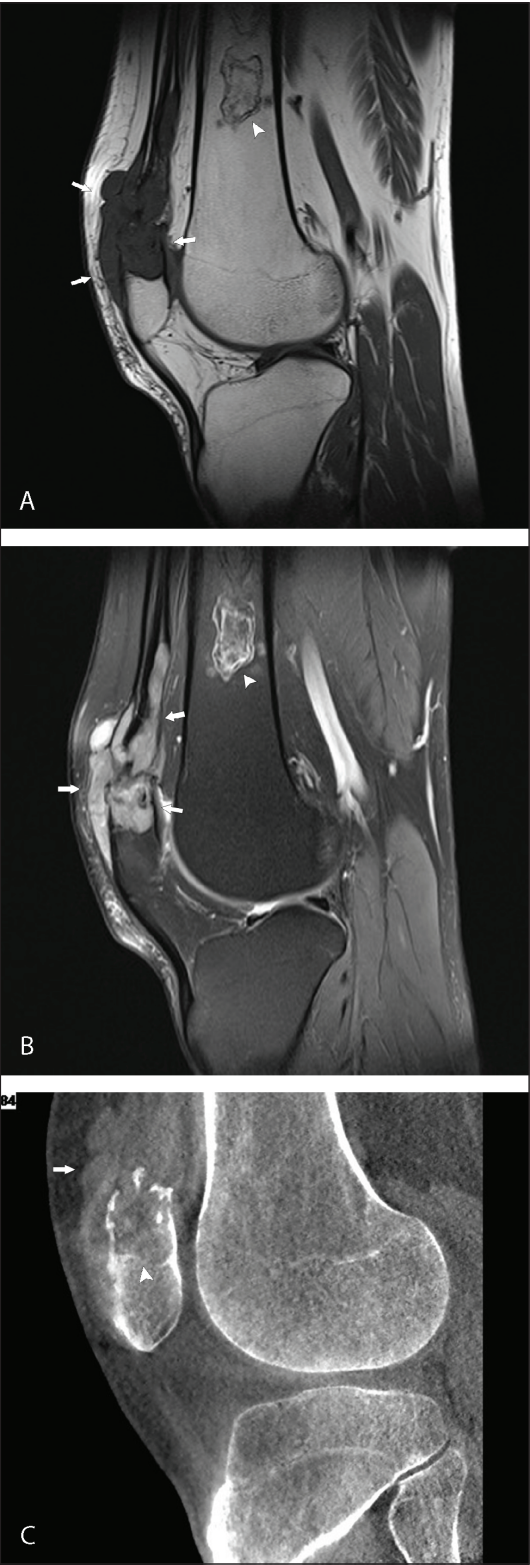


Based on the clinical presentation and imaging findings, tophaceous gout was suspected. After repeated anamnesis, the patient admitted alcohol overconsumption for many years and having previous gout attacks at the feet. Because of the aggressive nature of the process, a biopsy was performed in order to exclude a malignant tumor. The biopsy specimen showed the presence of monosodium urate (MSU) crystals, in keeping with tophaceous gout. The patient was reassured, was provided with appropriate dietary advice and nonsteroidal anti-inflammatory drugs.

## Comment

Gout is a metabolic disorder resulting in hyperuricemia. It is a very common condition with a peak incidence at the fifth decade of life. Men are more affected than women. Clinical manifestations of gout are asymmetric arthritis and/or soft tissue nodules. The most affected site is the first metatarsophalangeal joint, followed by the first interphalangeal joint. The hand and wrist are also commonly affected. The patella is an unusual site of gout. Clinical presentation and laboratory values are usually sufficient for diagnosis. The gold standard remains the demonstration of MSU crystals in the joint fluid or in the tophi.

The first stage in gout is an asymptomatic hyperuricemia without imaging abnormalities. After several years of hyperuricemia, a first attack of gouty arthritis can occur. This is usually monoarticular and radiographic evaluation remains normal. Sometimes only soft tissue swelling and joint effusion can be seen. Tophi and erosions occur usually more than 10 years after the first attack. This time is needed to store the MSU crystals in soft tissues.

Radiographic manifestations of chronic gout are well-defined punched-out erosions with overhanging edges, late preservation of the joint space, lack of periarticular bone demineralization, soft tissue nodules of high density, asymmetric involvement and intraosseous calcifications. Tophaceous gout is a term, designating the presence of MSU crystals within the soft tissues. Although relatively dense on plain films, soft tissue calcifications are very rare, unless there is decreased renal clearing of calcium in patients with renal insufficiency. Computed tomography (CT) and Magnetic Resonance Imaging (MRI) are usually not needed to diagnose gout and are nonspecific. On CT, tophi are slightly hyperdense compared to soft tissue. CT is more sensitive than plain films to detect subtle soft tissue calcifications even in the absence of renal insufficiency. Some authors recommend dual source CT for characterization of gout tophi. On MRI, tophi are hypointense on T1-WI and vary from hypointense to hyperintense on T2-WI. Heterogeneity on T2-WI may be seen, caused by urate crystals or calcium deposition. The pattern of enhancement is variable.

In most scenarios, gout is a straightforward diagnosis but can mimic infection (acute arthritis) or bone or soft tissue neoplasms if located at unusual sites, such as the quadriceps tendon and patella. The thought of it in the appropriate clinical setting enables the correct diagnosis. Additional clues are the knowledge of a history of gout at other joints, hyperuricemia, bone infarcts and alcohol abuse.

## Competing Interests

The authors declare that they have no competing interests.
